# Poly[[diaqua­bis(μ_3_-maleato-κ^4^ *O* ^1^:*O* ^1′^,*O* ^4^:O^4′^)dicopper(II)] trihydrate]

**DOI:** 10.1107/S1600536808023131

**Published:** 2008-07-26

**Authors:** Gregory A. Farnum, Robert L. LaDuca

**Affiliations:** a Lyman Briggs College, Department of Chemistry, Michigan State University, East Lansing, MI 48825, USA

## Abstract

In the title compound, {[Cu_2_(C_4_H_2_O_4_)_2_(H_2_O)_2_]·3H_2_O}_*n*_, Cu^II^ ions with square-pyramidal coordination are bridged by exo­tri­dentate maleate dianions into [Cu_2_(maleate)_2_(H_2_O)_2_]_*n*_ layers coincident with the *bc* crystal plane. The inter­lamellar regions contain hydrogen-bonded cyclic water hexa­mers which facilitate layer stacking into a pseudo-three-dimensional crystal structure.  The water hexamers themselves are formed by the operation of crystallographic inversion centers on sets of three crystallographically distinct water molecules of hydration.

## Related literature

For recent dpa coordination polymers, see: Brown *et al.* (2008 ▶). For the preparation of dpa, see: Zapf *et al.* (1998 ▶). For the determination of the τ factor for five-coordinate geometries, see: Addison *et al.* (1984 ▶). 
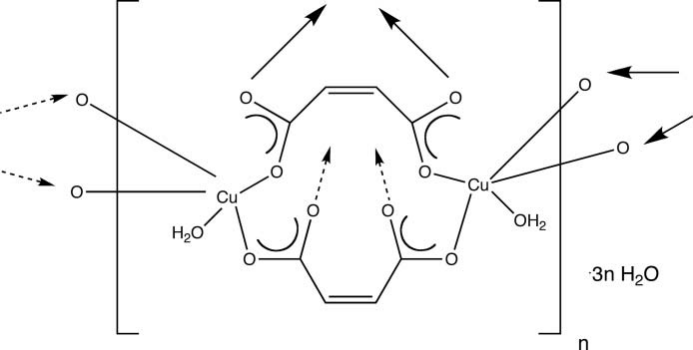


## Experimental

### 

#### Crystal data

[Cu_2_(C_4_H_2_O_4_)_2_(H_2_O)_2_]·3H_2_O
*M* *_r_* = 445.27
Monoclinic, 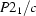

*a* = 8.8835 (14) Å
*b* = 8.7700 (14) Å
*c* = 18.814 (3) Å
β = 97.994 (3)°
*V* = 1451.5 (4) Å^3^
*Z* = 4
Mo *K*α radiation
μ = 3.00 mm^−1^
*T* = 173 (2) K
0.30 × 0.28 × 0.05 mm

#### Data collection

Bruker APEXII diffractometer
Absorption correction: multi-scan (*SADABS*; Sheldrick, 1996 ▶) *T*_min_ = 0.470, *T*_max_ = 0.860
9585 measured reflections
2643 independent reflections
2331 reflections with *I* > 2σ(*I*)
*R*_int_ = 0.026

#### Refinement

*R*[*F* ^2^ > 2σ(*F* ^2^)] = 0.023
*wR*(*F* ^2^) = 0.055
*S* = 1.03
2643 reflections
238 parameters
15 restraints
H atoms treated by a mixture of independent and constrained refinement
Δρ_max_ = 0.31 e Å^−3^
Δρ_min_ = −0.31 e Å^−3^

### 

Data collection: *COSMO* (Bruker, 2006 ▶); cell refinement: *APEX2* (Bruker, 2006 ▶); data reduction: *SAINT* (Bruker, 2006 ▶); program(s) used to solve structure: *SHELXS97* (Sheldrick, 2008 ▶); program(s) used to refine structure: *SHELXL97* (Sheldrick, 2008 ▶); molecular graphics: *CrystalMaker* (Palmer, 2007 ▶); software used to prepare material for publication: *SHELXL97*.

## Supplementary Material

Crystal structure: contains datablocks I, global. DOI: 10.1107/S1600536808023131/sj2520sup1.cif

Structure factors: contains datablocks I. DOI: 10.1107/S1600536808023131/sj2520Isup2.hkl

Additional supplementary materials:  crystallographic information; 3D view; checkCIF report

## Figures and Tables

**Table 1:** Hydrogen-bond geometry (Å, °)

*D*—H⋯*A*	*D*—H	H⋯*A*	*D*⋯*A*	*D*—H⋯*A*
O1*W*—H1*WA*⋯O5	0.878 (16)	2.034 (17)	2.910 (3)	175 (3)
O1*W*—H1*WB*⋯O2*W*	0.864 (16)	2.034 (18)	2.863 (3)	161 (3)
O2*W*—H2*WA*⋯O7	0.861 (16)	1.967 (17)	2.827 (3)	177 (3)
O2*W*—H2*WB*⋯O3*W*	0.851 (16)	2.014 (18)	2.854 (3)	169 (3)
O3*W*—H3*WA*⋯O2^i^	0.871 (16)	1.995 (19)	2.847 (2)	166 (3)
O3*W*—H3*WB*⋯O1*W*^i^	0.857 (16)	2.17 (2)	2.928 (3)	148 (2)
O9—H9*A*⋯O1*W*^ii^	0.853 (16)	1.987 (18)	2.831 (3)	170 (3)
O9—H9*B*⋯O10^iii^	0.851 (16)	2.023 (19)	2.855 (3)	165 (2)
O10—H10*A*⋯O2*W*^iv^	0.867 (16)	1.943 (17)	2.797 (3)	168 (3)
O10—H10*B*⋯O3*W*	0.846 (16)	2.059 (18)	2.879 (3)	163 (2)

Symmetry codes: (i) 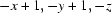; (ii) 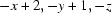; (iii) 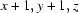; (iv) 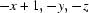.

**Fig. 1.**
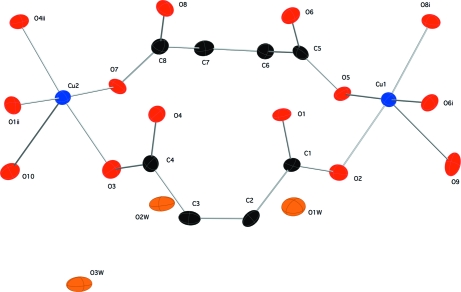
Asymmetric unit of the title compound, showing 50% probability ellipsoids and atom numbering scheme. Hydrogen atoms have been omitted. Color codes: blue Cu, red O within maleate moieties, orange O within water molecules, black C.

**Fig. 2.**
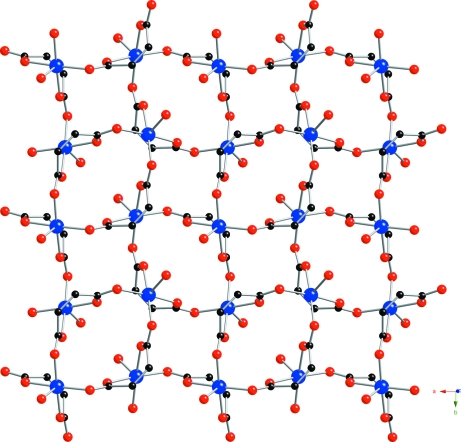
A single coordination polymer layer in the title compound, viewed down the c crystal direction.

**Fig. 3.**
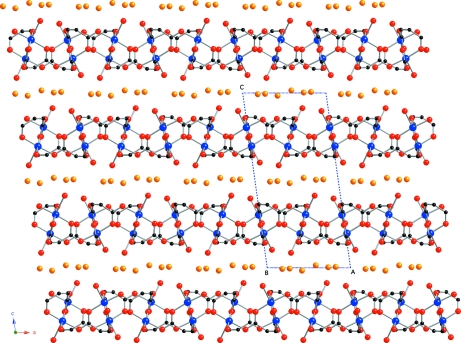
Packing diagram illustrating the ABAB layer stacking pattern, which forms the 3-D crystal structure of the title compound through hydrogen bonding between ligated and unligated water molecules.

**Fig. 4.**
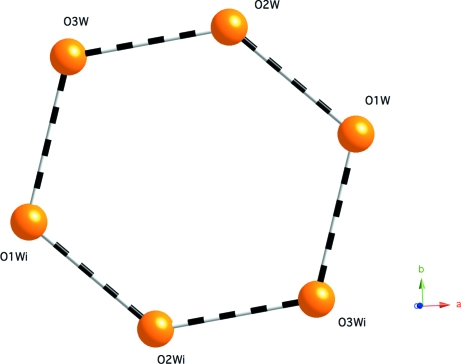
A single pseudo-planar cyclic water molecule hexamer in the title compound.

## supplementary crystallographic information

### Comment

Recently our group has been investigating metal dicarboxylate coordination polymers with 4,4'-dipyridylamine (dpa) co-ligands (Brown *et al.*, 2008). In an attempt to prepare a copper maleate/dpa dual-ligand coordination polymer, blue plates of the title compound were obtained. The asymmetric unit (Fig. 1) of the title compound contains two Cu^II^ ions, two maleate ligands and two aqua ligands along with three water molecules of crystallization. Each crystallographically distinct Cu^II^ ion manifests square pyramidal [CuO_5_] coordination with τ factors (Addison *et al.*, 1984) of 0.045 and 0.025 for Cu1 and Cu2, respectively.

Each Cu1 atom is connected to two Cu2 atoms by a exotridentate maleate ligand. In turn, each Cu2 atom is connected to two Cu1 atoms by a crystallographically distinct exotridentate maleate ligand. In this manner [Cu_2_(maleate)_2_(H_2_O)_2_]_n_ layers are constructed, coincident with the *bc* crystal planes (Fig. 2). The Cu atoms describe a (4,4) grid with Cu···Cu distances around the grid perimeter of 4.925 (1), 4.874 (1), 4.902 (1) and 4.835 (1) Å. The through-space Cu···Cu distances across the two different types of grid spaces measure 6.338 (1) and 6.261 (1) Å, and 5.390 and 7.094 Å.

Adjacent [Cu_2_(maleate)_2_(H_2_O)_2_]_n_ layers stack in an *ABAB* pattern to construct the three-dimensional crystal structure (Fig. 3) by means of O—H···O hydrogen bonding patterns between bound and unligated water molecules of crystallization. The unligated water molecules situated between the [Cu_2_(maleate)_2_(H_2_O)_2_]_n_ layers aggregate into *pseudo* co-planar cyclic hexamers by action of the crystallographic inversion centers on sets of three crystallographically distinct water molecules of hydration (Fig. 4).

### Experimental

Copper nitrate trihydrate and maleic acid were obtained commercially. 4,4'-dipyridylamine (dpa) was prepared *via* a published procedure (Zapf *et al.*, 1998). Copper nitrate trihydrate (17 mg, 0.07 mmol) and maleic acid (9 mg, 0.08 mmol) were dissolved in 1.5 ml water in a glass vial. A 0.75 ml aliquot of a 1:1 water:ethanol mixture was then added, followed by 1.5 ml of an ethanolic solution of dpa (32 mg, 0.19 mmol). Blue plates of the title compound deposited after standing at 25 °C for one week.

### Crystal data

**Table:**

[Cu_2_(C_4_H_2_O_4_)(H_2_O)_2_]·3H_2_O	*F*_000_ = 896
*M**_r_* = 445.27	*D*_x_ = 2.038 Mg m^−^^3^
Monoclinic, *P*2_1_/*c*	Mo *K*α radiation λ = 0.71073 Å
*a* = 8.8835 (14) Å	Cell parameters from 9585 reflections
*b* = 8.7700 (14) Å	θ = 2.2–25.3º
*c* = 18.814 (3) Å	µ = 3.00 mm^−^^1^
β = 97.994 (3)º	*T* = 173 (2) K
*V* = 1451.5 (4) Å^3^	Plate, blue
*Z* = 4	0.30 × 0.28 × 0.05 mm

### Data collection

**Table:**

Bruker APEXII diffractometer	2643 independent reflections
Radiation source: fine-focus sealed tube	2331 reflections with *I* > 2σ(*I*)
Monochromator: graphite	*R*_int_ = 0.026
*T* = 173(2) K	θ_max_ = 25.3º
ω/ψ scans	θ_min_ = 2.2º
Absorption correction: multi-scan(SADABS; Sheldrick, 1996)	*h* = −7→10
*T*_min_ = 0.471, *T*_max_ = 0.860	*k* = −10→10
9585 measured reflections	*l* = −22→21

### Refinement

**Table:**

Refinement on *F*^2^	Secondary atom site location: difference Fourier map
Least-squares matrix: full	Hydrogen site location: inferred from neighbouring sites
*R*[*F*^2^ > 2σ(*F*^2^)] = 0.023	H atoms treated by a mixture of independent and constrained refinement
*wR*(*F*^2^) = 0.055	*w* = 1/[σ^2^(*F*_o_^2^) + (0.0212*P*)^2^ + 1.6998*P*] where *P* = (*F*_o_^2^ + 2*F*_c_^2^)/3
*S* = 1.03	(Δ/σ)_max_ = 0.001
2643 reflections	Δρ_max_ = 0.31 e Å^−^^3^
238 parameters	Δρ_min_ = −0.31 e Å^−^^3^
15 restraints	Extinction correction: none
Primary atom site location: structure-invariant direct methods	

### Special details

**Table:**

Geometry. All e.s.d.'s (except the e.s.d. in the dihedral angle between two l.s. planes) are estimated using the full covariance matrix. The cell e.s.d.'s are taken into account individually in the estimation of e.s.d.'s in distances, angles and torsion angles; correlations between e.s.d.'s in cell parameters are only used when they are defined by crystal symmetry. An approximate (isotropic) treatment of cell e.s.d.'s is used for estimating e.s.d.'s involving l.s. planes.
Refinement. Refinement of *F*^2^ against ALL reflections. The weighted *R*-factor *wR* and goodness of fit *S* are based on *F*^2^, conventional *R*-factors *R* are based on *F*, with *F* set to zero for negative *F*^2^. The threshold expression of *F*^2^ > σ(*F*^2^) is used only for calculating *R*-factors(gt) *etc*. and is not relevant to the choice of reflections for refinement. *R*-factors based on *F*^2^ are statistically about twice as large as those based on *F*, and *R*- factors based on ALL data will be even larger.

### Fractional atomic coordinates and isotropic or equivalent isotropic displacement parameters (Å^2^)

**Table:**

	*x*	*y*	*z*	*U*_iso_*/*U*_eq_	
Cu1	1.01597 (3)	0.64409 (3)	0.197428 (15)	0.01156 (9)	
Cu2	0.45106 (3)	0.07186 (3)	0.195099 (15)	0.01174 (9)	
O1	0.72160 (19)	0.60380 (19)	0.25258 (8)	0.0151 (4)	
O1W	0.8228 (2)	0.4259 (2)	0.00615 (10)	0.0233 (4)	
H1WA	0.886 (3)	0.433 (3)	0.0464 (12)	0.028*	
H1WB	0.746 (2)	0.374 (3)	0.0165 (14)	0.028*	
O2	0.80468 (19)	0.6770 (2)	0.15230 (9)	0.0156 (4)	
O2W	0.5879 (2)	0.2061 (2)	0.01524 (10)	0.0225 (4)	
H2WA	0.605 (3)	0.157 (3)	0.0552 (11)	0.027*	
H2WB	0.495 (2)	0.233 (3)	0.0091 (14)	0.027*	
O3	0.4171 (2)	0.27085 (19)	0.14892 (9)	0.0164 (4)	
O3W	0.2668 (2)	0.2526 (2)	−0.00371 (10)	0.0236 (4)	
H3WA	0.238 (3)	0.258 (3)	−0.0498 (9)	0.028*	
H3WB	0.242 (3)	0.338 (2)	0.0134 (13)	0.028*	
O4	0.5053 (2)	0.38423 (19)	0.25143 (9)	0.0150 (4)	
O5	1.0151 (2)	0.44986 (19)	0.14409 (9)	0.0152 (4)	
O6	0.9918 (2)	0.32879 (19)	0.24548 (9)	0.0148 (4)	
O7	0.63415 (19)	0.0476 (2)	0.14736 (9)	0.0152 (4)	
O8	0.78378 (19)	0.09818 (19)	0.24870 (9)	0.0151 (4)	
O9	1.0870 (2)	0.7605 (2)	0.10332 (9)	0.0191 (4)	
H9A	1.103 (3)	0.701 (3)	0.0691 (12)	0.023*	
H9B	1.149 (3)	0.835 (2)	0.1052 (14)	0.023*	
O10	0.3104 (2)	−0.0132 (2)	0.08656 (9)	0.0162 (4)	
H10A	0.355 (3)	−0.071 (2)	0.0582 (13)	0.019*	
H10B	0.280 (3)	0.066 (2)	0.0636 (13)	0.019*	
C1	0.6980 (3)	0.6406 (3)	0.18741 (13)	0.0132 (5)	
C2	0.5430 (3)	0.6438 (3)	0.14606 (13)	0.0129 (5)	
H2	0.5162	0.7317	0.1176	0.016*	
C3	0.4383 (3)	0.5354 (3)	0.14503 (13)	0.0140 (5)	
H3	0.3437	0.5533	0.1160	0.017*	
C4	0.4550 (3)	0.3888 (3)	0.18500 (13)	0.0143 (5)	
C5	1.0001 (3)	0.3283 (3)	0.17929 (13)	0.0131 (5)	
C6	0.9934 (3)	0.1841 (3)	0.13696 (13)	0.0135 (5)	
H6	1.0682	0.1703	0.1060	0.016*	
C7	0.8919 (3)	0.0730 (3)	0.13873 (13)	0.0142 (5)	
H7	0.9019	−0.0140	0.1097	0.017*	
C8	0.7639 (3)	0.0732 (3)	0.18235 (13)	0.0133 (5)	

### Atomic displacement parameters (Å^2^)

**Table:**

	*U*^11^	*U*^22^	*U*^33^	*U*^12^	*U*^13^	*U*^23^
Cu1	0.01167 (17)	0.01221 (16)	0.01043 (16)	−0.00015 (13)	0.00021 (12)	−0.00049 (12)
Cu2	0.01275 (18)	0.01194 (16)	0.01041 (16)	−0.00027 (13)	0.00118 (12)	−0.00011 (12)
O1	0.0139 (10)	0.0193 (9)	0.0121 (9)	−0.0001 (8)	0.0017 (7)	0.0033 (7)
O1W	0.0287 (12)	0.0259 (11)	0.0142 (10)	−0.0018 (9)	−0.0006 (8)	0.0005 (9)
O2	0.0113 (9)	0.0225 (10)	0.0129 (9)	−0.0018 (8)	0.0013 (7)	0.0026 (7)
O2W	0.0208 (11)	0.0305 (11)	0.0160 (10)	0.0059 (9)	0.0017 (8)	0.0050 (9)
O3	0.0251 (11)	0.0108 (9)	0.0127 (9)	−0.0018 (8)	0.0007 (7)	−0.0023 (7)
O3W	0.0308 (12)	0.0228 (10)	0.0156 (10)	0.0031 (9)	−0.0026 (9)	−0.0006 (8)
O4	0.0192 (10)	0.0127 (9)	0.0125 (9)	−0.0007 (8)	0.0004 (7)	0.0007 (7)
O5	0.0186 (10)	0.0116 (9)	0.0148 (9)	−0.0017 (8)	−0.0002 (7)	0.0018 (7)
O6	0.0191 (10)	0.0121 (9)	0.0133 (9)	−0.0004 (7)	0.0024 (7)	0.0009 (7)
O7	0.0086 (9)	0.0219 (10)	0.0143 (9)	−0.0009 (8)	−0.0008 (7)	0.0002 (8)
O8	0.0127 (10)	0.0190 (9)	0.0136 (9)	−0.0008 (8)	0.0013 (7)	−0.0008 (7)
O9	0.0230 (11)	0.0158 (10)	0.0203 (10)	−0.0052 (8)	0.0096 (8)	−0.0013 (8)
O10	0.0181 (10)	0.0148 (10)	0.0160 (10)	0.0018 (8)	0.0031 (8)	−0.0004 (8)
C1	0.0158 (14)	0.0088 (12)	0.0149 (13)	0.0013 (11)	0.0019 (11)	−0.0024 (10)
C2	0.0149 (14)	0.0131 (13)	0.0108 (12)	0.0026 (11)	0.0018 (10)	0.0013 (10)
C3	0.0141 (14)	0.0157 (13)	0.0114 (12)	0.0058 (11)	−0.0005 (10)	−0.0007 (10)
C4	0.0089 (13)	0.0168 (13)	0.0180 (14)	0.0003 (11)	0.0050 (10)	0.0002 (11)
C5	0.0075 (13)	0.0148 (13)	0.0162 (14)	0.0011 (10)	−0.0013 (10)	−0.0003 (11)
C6	0.0124 (13)	0.0133 (13)	0.0156 (13)	0.0034 (11)	0.0046 (10)	0.0009 (10)
C7	0.0159 (14)	0.0122 (12)	0.0144 (13)	0.0045 (11)	0.0021 (10)	−0.0002 (10)
C8	0.0167 (14)	0.0074 (12)	0.0157 (13)	0.0017 (11)	0.0018 (11)	0.0012 (10)

### Geometric parameters (Å, °)

**Table:**

Cu1—O6^i^	1.9501 (17)	O4—Cu2^iii^	1.9395 (17)
Cu1—O8^i^	1.9628 (17)	O5—C5	1.272 (3)
Cu1—O2	1.9708 (17)	O6—C5	1.258 (3)
Cu1—O5	1.9765 (17)	O6—Cu1^iv^	1.9501 (17)
Cu1—O9	2.2101 (17)	O7—C8	1.265 (3)
Cu2—O4^ii^	1.9395 (17)	O8—C8	1.255 (3)
Cu2—O3	1.9541 (17)	O8—Cu1^iv^	1.9627 (17)
Cu2—O1^ii^	1.9544 (17)	O9—H9A	0.853 (16)
Cu2—O7	1.9757 (17)	O9—H9B	0.851 (16)
Cu2—O10	2.3618 (18)	O10—H10A	0.867 (16)
O1—C1	1.257 (3)	O10—H10B	0.846 (16)
O1—Cu2^iii^	1.9544 (17)	C1—C2	1.485 (3)
O1W—H1WA	0.878 (16)	C2—C3	1.328 (4)
O1W—H1WB	0.864 (16)	C2—H2	0.9500
O2—C1	1.269 (3)	C3—C4	1.487 (3)
O2W—H2WA	0.861 (16)	C3—H3	0.9500
O2W—H2WB	0.851 (16)	C5—C6	1.492 (3)
O3—C4	1.257 (3)	C6—C7	1.331 (4)
O3W—H3WA	0.871 (16)	C6—H6	0.9500
O3W—H3WB	0.857 (16)	C7—C8	1.492 (3)
O4—C4	1.268 (3)	C7—H7	0.9500
			
O6^i^—Cu1—O8^i^	89.13 (7)	Cu1—O9—H9A	114.8 (18)
O6^i^—Cu1—O2	90.64 (7)	Cu1—O9—H9B	125.1 (18)
O8^i^—Cu1—O2	173.22 (7)	H9A—O9—H9B	109 (2)
O6^i^—Cu1—O5	176.01 (7)	Cu2—O10—H10A	118.9 (19)
O8^i^—Cu1—O5	91.41 (7)	Cu2—O10—H10B	105.9 (18)
O2—Cu1—O5	88.35 (7)	H10A—O10—H10B	108 (2)
O6^i^—Cu1—O9	95.37 (7)	O1—C1—O2	122.5 (2)
O8^i^—Cu1—O9	99.75 (7)	O1—C1—C2	122.2 (2)
O2—Cu1—O9	87.02 (7)	O2—C1—C2	115.3 (2)
O5—Cu1—O9	88.44 (7)	C3—C2—C1	126.2 (2)
O4^ii^—Cu2—O3	174.68 (7)	C3—C2—H2	116.9
O4^ii^—Cu2—O1^ii^	88.55 (7)	C1—C2—H2	116.9
O3—Cu2—O1^ii^	90.71 (7)	C2—C3—C4	126.3 (2)
O4^ii^—Cu2—O7	91.53 (7)	C2—C3—H3	116.9
O3—Cu2—O7	88.85 (7)	C4—C3—H3	116.9
O1^ii^—Cu2—O7	176.09 (7)	O3—C4—O4	122.5 (2)
O4^ii^—Cu2—O10	102.95 (7)	O3—C4—C3	116.0 (2)
O3—Cu2—O10	82.37 (7)	O4—C4—C3	121.5 (2)
O1^ii^—Cu2—O10	97.06 (7)	O6—C5—O5	122.5 (2)
O7—Cu2—O10	86.73 (7)	O6—C5—C6	121.9 (2)
C1—O1—Cu2^iii^	119.48 (16)	O5—C5—C6	115.6 (2)
H1WA—O1W—H1WB	106 (2)	C7—C6—C5	125.7 (2)
C1—O2—Cu1	118.31 (16)	C7—C6—H6	117.1
H2WA—O2W—H2WB	108 (2)	C5—C6—H6	117.1
C4—O3—Cu2	118.78 (16)	C6—C7—C8	125.7 (2)
H3WA—O3W—H3WB	106 (2)	C6—C7—H7	117.1
C4—O4—Cu2^iii^	120.15 (16)	C8—C7—H7	117.1
C5—O5—Cu1	116.82 (16)	O8—C8—O7	122.7 (2)
C5—O6—Cu1^iv^	123.65 (16)	O8—C8—C7	122.3 (2)
C8—O7—Cu2	119.51 (16)	O7—C8—C7	115.0 (2)
C8—O8—Cu1^iv^	122.77 (16)		
			
O6^i^—Cu1—O2—C1	−78.93 (18)	O2—C1—C2—C3	132.4 (3)
O8^i^—Cu1—O2—C1	9.1 (7)	C1—C2—C3—C4	−0.1 (4)
O5—Cu1—O2—C1	97.21 (18)	Cu2—O3—C4—O4	−4.7 (3)
O9—Cu1—O2—C1	−174.27 (18)	Cu2—O3—C4—C3	174.94 (16)
O4^ii^—Cu2—O3—C4	−6.9 (9)	Cu2^iii^—O4—C4—O3	−175.98 (18)
O1^ii^—Cu2—O3—C4	75.07 (18)	Cu2^iii^—O4—C4—C3	4.4 (3)
O7—Cu2—O3—C4	−101.05 (18)	C2—C3—C4—O3	−130.6 (3)
O10—Cu2—O3—C4	172.09 (19)	C2—C3—C4—O4	49.0 (4)
O6^i^—Cu1—O5—C5	−27.4 (11)	Cu1^iv^—O6—C5—O5	−175.24 (17)
O8^i^—Cu1—O5—C5	70.43 (17)	Cu1^iv^—O6—C5—C6	4.5 (3)
O2—Cu1—O5—C5	−102.78 (17)	Cu1—O5—C5—O6	−2.6 (3)
O9—Cu1—O5—C5	170.15 (18)	Cu1—O5—C5—C6	177.60 (16)
O4^ii^—Cu2—O7—C8	−74.33 (18)	O6—C5—C6—C7	47.7 (4)
O3—Cu2—O7—C8	100.36 (18)	O5—C5—C6—C7	−132.5 (3)
O1^ii^—Cu2—O7—C8	16.7 (11)	C5—C6—C7—C8	1.2 (4)
O10—Cu2—O7—C8	−177.22 (18)	Cu1^iv^—O8—C8—O7	−178.04 (17)
Cu2^iii^—O1—C1—O2	173.13 (17)	Cu1^iv^—O8—C8—C7	1.3 (3)
Cu2^iii^—O1—C1—C2	−7.1 (3)	Cu2—O7—C8—O8	7.6 (3)
Cu1—O2—C1—O1	9.6 (3)	Cu2—O7—C8—C7	−171.73 (15)
Cu1—O2—C1—C2	−170.14 (16)	C6—C7—C8—O8	−52.5 (4)
O1—C1—C2—C3	−47.3 (4)	C6—C7—C8—O7	126.8 (3)

Symmetry codes: (i) −*x*+2, *y*+1/2, −*z*+1/2; (ii) −*x*+1, *y*−1/2, −*z*+1/2; (iii) −*x*+1, *y*+1/2, −*z*+1/2; (iv) −*x*+2, *y*−1/2, −*z*+1/2.

### Hydrogen-bond geometry (Å, °)

**Table:**

*D*—H···*A*	*D*—H	H···*A*	*D*···*A*	*D*—H···*A*
O1W—H1WA···O5	0.878 (16)	2.034 (17)	2.910 (3)	175 (3)
O1W—H1WB···O2W	0.864 (16)	2.034 (18)	2.863 (3)	161 (3)
O2W—H2WA···O7	0.861 (16)	1.967 (17)	2.827 (3)	177 (3)
O2W—H2WB···O3W	0.851 (16)	2.014 (18)	2.854 (3)	169 (3)
O3W—H3WA···O2^v^	0.871 (16)	1.995 (19)	2.847 (2)	166 (3)
O3W—H3WB···O1W^v^	0.857 (16)	2.17 (2)	2.928 (3)	148 (2)
O9—H9A···O1W^vi^	0.853 (16)	1.987 (18)	2.831 (3)	170 (3)
O9—H9B···O10^vii^	0.851 (16)	2.023 (19)	2.855 (3)	165 (2)
O10—H10A···O2W^viii^	0.867 (16)	1.943 (17)	2.797 (3)	168 (3)
O10—H10B···O3W	0.846 (16)	2.059 (18)	2.879 (3)	163 (2)

Symmetry codes: (v) −*x*+1, −*y*+1, −*z*; (vi) −*x*+2, −*y*+1, −*z*; (vii) *x*+1, *y*+1, *z*; (viii) −*x*+1, −*y*, −*z*.
